# SLC35A2 Deficiency Promotes an Epithelial-to-Mesenchymal Transition-like Phenotype in Madin–Darby Canine Kidney Cells

**DOI:** 10.3390/cells11152273

**Published:** 2022-07-23

**Authors:** Magdalena Kot, Ewa Mazurkiewicz, Maciej Wiktor, Wojciech Wiertelak, Antonina Joanna Mazur, Andrei Rahalevich, Mariusz Olczak, Dorota Maszczak-Seneczko

**Affiliations:** 1 Department of Cell Pathology, Faculty of Biotechnology, University of Wroclaw, 14A F. Joliot-Curie St., 50-383 Wroclaw, Poland; magdalena.kot@uwr.edu.pl (M.K.); ewa.mazurkiewicz@uwr.edu.pl (E.M.); antonina.mazur@uwr.edu.pl (A.J.M.); 2 Department of Biochemistry, Faculty of Biotechnology, University of Wroclaw, 14A F. Joliot-Curie St., 50-383 Wroclaw, Poland; maciej.wiktor@uwr.edu.pl (M.W.); wojciech.wiertelak@uwr.edu.pl (W.W.); andrrag2000@gmail.com (A.R.); mariusz.olczak@uwr.edu.pl (M.O.)

**Keywords:** solute carrier family 35 member A2 (SLC35A2), Madin–Darby canine kidney (MDCK) cells, epithelial-to-mesenchymal transition (EMT), UDP-galactose, glycosylation, Golgi complex, congenital disorder of glycosylation (CDG), fibronectin, vimentin, migration

## Abstract

In mammalian cells, SLC35A2 delivers UDP–galactose for galactosylation reactions that take place predominantly in the Golgi lumen. Mutations in the corresponding gene cause a subtype of a congenital disorder of glycosylation (SLC35A2-CDG). Although more and more patients are diagnosed with SLC35A2-CDG, the link between defective galactosylation and disease symptoms is not fully understood. According to a number of reports, impaired glycosylation may trigger the process of epithelial-to-mesenchymal transition (EMT). We therefore examined whether the loss of SLC35A2 activity would promote EMT in a non-malignant epithelial cell line. For this purpose, we knocked out the *SLC35A2* gene in Madin–Darby canine kidney (MDCK) cells. The resulting clones adopted an elongated, spindle-shaped morphology and showed impaired cell–cell adhesion. Using qPCR and western blotting, we revealed down-regulation of E-cadherin in the knockouts, while the fibronectin and vimentin levels were elevated. Moreover, the knockout cells displayed reorganization of vimentin intermediate filaments and altered subcellular distribution of a vimentin-binding protein, formiminotransferase cyclodeaminase (FTCD). Furthermore, depletion of SLC35A2 triggered Golgi compaction. Finally, the *SLC35A2* knockouts displayed increased motility and invasiveness. In conclusion, SLC35A2-deficient MDCK cells showed several hallmarks of EMT. Our findings point to a novel role for SLC35A2 as a gatekeeper of the epithelial phenotype.

## 1. Introduction

Glycosylation is one of the most important modifications of proteins and lipids in eukaryotic cells. Glycoconjugates play fundamental roles in the growth and development of multicellular organisms, as well as in numerous molecular recognition events. Alterations in glycosylation can modulate inflammatory responses, enable viral immune escape, and promote cancer cell metastasis [1].

The main classes of glycoconjugates include glycoproteins, glycolipids, and proteoglycans. They all contain at least one oligosaccharide chain, i.e., glycan, covalently attached to a non-carbohydrate part. In glycoproteins, glycans are attached to proteins via two types of linkages: N-glycans are attached to Asn residues present in a canonical consensus sequence Asn-X-Ser/Thr, where X can be any amino acid except Pro, whereas O-glycans are linked to either Ser or Thr residues. In glycolipids, glycans are covalently joined to a ceramide backbone. Finally, proteoglycans consist of core proteins with one or more covalently attached glycosaminoglycan (GAG) chains.

The majority of glycoconjugates are synthesized in the endoplasmic reticulum (ER) and Golgi lumen by glycosyltransferases. Most of these enzymes are type II membrane proteins with a short N-terminal cytoplasmic tail, a single transmembrane domain, and a large globular C-terminal catalytic domain facing the lumen of the organelle [2]. Glycosyltransferases use activated forms of monosaccharides (nucleotide sugars) as substrates. These compounds are synthesized outside the ER/Golgi lumen and subsequently translocated across organelle membranes by nucleotide sugar transporters (NSTs) belonging to the solute carrier 35 (SLC35) family of proteins [3]. NSTs are multispanning proteins with a molecular weight of ~30–45 kDa, an even number of transmembrane domains, and cytosolic orientation of the N- and C-termini. They are considered to act as antiporters that exchange the nucleotide sugar molecule for the corresponding nucleoside monophosphate (the latter is formed upon glycosylation and dephosphorylation reactions).

Galactose is an aldohexose monosaccharide and a structural isomer of glucose. It is a component of all main classes of glycosylated macromolecules, i.e., glycoproteins, glycolipids, and proteoglycans. In mammalian glycoconjugates, galactose either occupies terminal or subterminal position (e.g., in N-glycans or lactosylceramide) or is located internally (e.g., in the canonical tetrasaccharide that joins GAG chains to core proteins in proteoglycans). Incorporation of galactose into glycoconjugates is mediated by a number of galactosyltransferases, which utilize uridine diphosphate galactose (UDP-galactose) as a sugar donor. UDP-galactose is synthesized in the cytoplasm by the enzymes of the Leloir pathway, whereas the catalytic centers of the majority of galactosyltransferases are facing the Golgi lumen, which necessitates the existence of a dedicated transport system in the Golgi membranes.

Delivery of UDP-galactose into the Golgi lumen is mediated by the UDP-galactose transporter (UGT; SLC35A2) encoded by the X-linked *SLC35A2* gene. In mammals, two SLC35A2 splice variants have been identified: a Golgi-resident UGT1 and UGT2, which localizes to both the ER and Golgi apparatus and contains the C-terminal ER retaining dilysine motif (KVKGS), which is not present in UGT1 [4]. The subcellular localization of both variants and their involvement in galactosylation of macromolecules was most extensively studied in the Chinese hamster ovary (CHO) and Madin–Darby canine kidney (MDCK) cell lines [5,6,7]. Glycoconjugates synthesized by the mutant cell lines deficient in the SLC35A2 activity are severely undergalactosylated [e.g., [7,8,9,10,11]], which demonstrates that this protein is indispensable for proper galactosylation of macromolecules.

A number of SLC35A2 interaction partners have been identified to date, including its kins SLC35A3 [12,13] and SLC35A5 [14], some functionally related glycosyltransferases [15,16,17,18], and proteins involved in the pH and ion homeostasis of the Golgi complex [19]. These results suggest that SLC35A2 does not act independently but rather interacts with numerous proteins in order to fulfill its function. On the other hand, some of the newly identified interaction partners of SLC35A2 (e.g., ATPases and ion transporters) suggest that its role(s) may extend beyond UDP-galactose delivery into the Golgi lumen.

Congenital disorders of glycosylation (CDGs) are a large and heterogenous group of rare genetic metabolic diseases caused by defects in glycan synthesis and/or modification pathways [20]. To date, more than 130 CDG subtypes have been characterized. The majority of CDGs are autosomal recessive in inheritance, although autosomal dominant as well as X-linked forms have also been reported [21]. The clinical manifestations of CDGs are very diverse and the most commonly occurring symptoms include developmental retardation, failure to thrive, hypotonia, neurological problems, hepatopathy, and coagulopathy [21].

For certain CDG subtypes, the relationship between the glycosylation defect and the disease symptoms is well-established. In SLC35C1-CDG, for example, selectin ligands on leukocytes are significantly underfucosylated due to compromised activity of the Golgi GDP–fucose transporter [22]. This prevents tethering and rolling of leukocytes on vascular endothelium which ultimately attenuates inflammatory response. However, for many CDGs, the influence of defective glycosylation on the downstream cellular phenotypes is poorly understood. 

Mutations in the *SLC35A2* gene are also a cause of a CDG subtype (SLC35A2-CDG; CDG IIm) [e.g., [23,24,25,26,27,28]]. The affected individuals usually experience neurological problems (global developmental delay, epilepsy, encephalopathy), as well as hypotonia. A number of SLC35A2-CDG patients fail to thrive due to gastrointestinal disease and impairment of the growth hormone–insulin-like growth factor axis [29]. They also show dysfunctions of the liver, spleen, kidney, and skeleton. However, it is not clear how an impaired Golgi UDP-galactose transporting activity mechanistically contributes to pathophysiology and clinical manifestation of SLC35A2-CDG.

An epithelial-to-mesenchymal transition (EMT) is a biological process during which epithelial cells undergo multiple biochemical changes that lead to the acquisition of a mesenchymal phenotype characterized by an enhanced motility and invasiveness as well as increased production of extracellular matrix (ECM) components [30]. EMT was first described by Hay in 1995 [31]. It is important for embryonic development as it allows the formation of tissues and organs [32]. Furthermore, it plays a role in the wound-healing process. However, an unwanted EMT may lead to a number of pathological changes, such as carcinogenesis or fibrosis. Apart from altered cellular morphology and increased migratory properties, EMT is accompanied by a unique gene expression signature. Specifically, cells undergoing EMT lose their major cell–cell adhesion molecule E-cadherin, whereas the fibronectin, vimentin, and N-cadherin levels become elevated [30].

EMT is regulated by a number of transcription factors including Snail, ZEB, and helix-loop-helix (HLH) family members, as well as some signaling pathways involving, e.g., transforming growth factor β (TGF-β) [32]. The latter is commonly used to induce EMT in experimental conditions. Interestingly, glycosylation alterations are also associated with EMT [33]. It has to be emphasized that the nature of the link between glycosylation and EMT is bidirectional, as not only EMT drives specific changes in the cellular glycosylation patterns, but it can also be triggered by an altered glycosylation.

Although it is known that the SLC35A2 activity is required for proper glycosylation, so far it has not been investigated whether its depletion triggers EMT. In this study, we knocked out the *SLC35A2* gene in the non-malignant epithelial MDCK cell line using the CRISPR/Cas9 strategy and found that it promoted several mesenchymal traits in the resulting clones. The changes included downregulation of E-cadherin expression and upregulation of fibronectin expression, as well as elevated level of its secretion, upregulation of vimentin expression, reorganization of vimentin cytoskeleton, Golgi compaction, and, finally, increased cell motility and invasiveness. Based on our findings, we suggest the possibility of a pathological, excessive EMT occurring in tissues of SLC35A2-CDG patients. The results obtained in this study highlight the importance of SLC35A2 for the maintenance of the epithelial characteristics of the cells.

## 2. Materials and Methods

### 2.1. Cell Culture Maintenance

MDCK II cell line was obtained from the European Collection of Authenticated Cell Cultures (ECACC). The wild-type and *SLC35A2* knockout cells were grown in Minimal Essential Medium Eagle (MEM) supplemented with fetal bovine serum (10%), penicillin (100 U/mL), and streptomycin (100 μg/mL) under standard conditions (37 °C, 5% CO_2_). The cells were passaged three times a week. Cellular morphology was documented with a Leica DMI 3000 B microscope (Leica Microsystems, Wetzlar, Germany).

### 2.2. CRISPR/Cas9-Assisted Gene Inactivation

SLC35A2-deficient MDCK clones were generated using a commercial CRISPR/Cas9 gene editing system (Dharmacon, Lafayette, CO, USA). Three crRNAs targeting different regions of the canine *SLC35A2* gene were used (5′-AGCCGCCGCGTTGGAACCGC-3′, 5′-GGAGCCTGGAACCGCTAGTG-3′ and 5′-GGGATATGTATTTGAGGCGC-3′).

The wild-type cells were co-transfected with the crRNA and tracrRNA complexes and a Cas9-encoding plasmid according to the manufacturer’s instructions. Initial enrichment for gene-edited cells was performed by growing transfected cells for three days in a complete medium supplemented with puromycin (1 µg/mL). Further enrichment involved selection on phytohemagglutinin-L (PHA-L, Vector Laboratories, Burlingame, CA, USA) and was achieved by culturing the cells for two weeks in a complete medium supplemented with 20 µg/mL of PHA-L (PHA-L is a toxic plant lectin that specifically binds to galactosylated β-1,6-branched complex-type N-glycans exposed on the surface of the wild-type cells, whereas the SLC35A2-deficient cells are not targeted by PHA-L and thus preferentially survive). 

After selection on PHA-L, clones were isolated and primarily screened via labeling of cell surface with biotinylated *Griffonia simplicifolia* lectin II (GSL II; Vector Laboratories, Burlingame, CA, USA) followed by a fluorophore-conjugated streptavidin. The SLC35A2 knockout in the selected clones was ultimately confirmed using immunostaining with a rabbit anti-SLC35A2 antibody targeting the C-terminus of the protein (Table S1) followed by a fluorophore-conjugated secondary antibody. Moreover, the genomic DNA was isolated from putative knockout clones and sequenced in order to confirm the absence of the wild-type *SLC35A2* sequence.

### 2.3. Immunofluorescence and Fluorescence Staining

For immunostaining of intracellular antigens, the cells were seeded onto 8-well microscope slides (Merck, Darmstadt, Germany) in a complete medium and grown for 24 h. For immunostaining of SLC35A2, GM130, and γ-adaptin, cells were treated as described previously [34]. The same procedure was used for fluorescence staining of filamentous actin (F-actin). For immunostaining of vimentin and FTCD, cells were fixed and permeabilized with ice-cold methanol for 10 min followed by blocking in 10% normal goat serum in PBS. Incubations with primary and secondary antibodies were performed in 10% normal goat serum in PBS. The antibodies used for immunofluorescence stainings are listed in Supplementary Table S1. The slides were imaged with a Leica SP8 confocal microscope (Leica Microsystems, Wetzlar, Germany).

### 2.4. Fluorescence Lectin Staining

For lectin staining, the cells were seeded, fixed, and permeabilized as described previously [34]. Next, cells were incubated for 1 h with biotinylated lectins (GSL II and VVL, Vector Laboratories, Burlingame, CA, USA) diluted 1:100 in the blocking solution (1% BSA with 0.1% saponin in PBS), followed by incubation for 1 h with streptavidin conjugated with Alexa Fluor 488 (Thermo Fisher Scientific, Waltham, MA, USA) diluted 1:200 in the blocking solution. The slides were imaged with a Leica SP8 confocal microscope (Leica Microsystems, Wetzlar, Germany).

### 2.5. In Situ Proximity Ligation Assay (PLA)

In situ PLA experiments were performed using Duolink reagents (Sigma-Aldrich, St. Louis, MO, USA) according to the manufacturer’s instructions. The cells were seeded on 8-well microscope slides in a complete medium and grown for 24 h. Next, cells were fixed with 4% paraformaldehyde in PBS for 20 min at room temperature (RT) and permeabilized for 5 min using 0.02% Triton X-100 in PBS at RT. Nonspecific binding sites were blocked with 3% BSA in PBS for 1 h at RT. After blocking, slides were incubated overnight at 4 °C with a 1:25 dilution of a mouse anti-FTCD antibody (Table S1) and a 1:100 dilution of a rabbit anti-vimentin antibody (H-84, Santa Cruz Biotechnology, Dallas, TX, USA). Next, cells were incubated with anti-mouse PLA probe PLUS and anti-rabbit PLA probe MINUS (both diluted 1:5 in 3% BSA in PBS) for 1 h at 37 °C. Between all steps, as well as before permeabilization, slides were washed with PBS three times for 5 min. After the incubation with PLA probes, cells were washed twice with 1X concentrated Buffer A for 5 min and incubated with a ligation solution for 30 min at 37 °C. Next, slides were washed twice with 1X concentrated buffer A for 2 min and incubated with Amplification Solution (Detection Reagents Orange, λ_ex_ = 554 nm and λ_em_ = 576 nm) for 100 min at 37 °C. Finally, slides were washed twice with 1X concentrated buffer B for 10 min and once with 0.01X concentrated buffer B for 1 min, mounted onto glass coverslips using Mounting Medium with DAPI, and imaged with a Zeiss LSM510 confocal microscope (Carl Zeiss, Jena, Germany).

### 2.6. Western Blotting and Lectin Blotting

For detection of intracellular or plasma membrane proteins, the cells were lysed and proteins present in cell lysates were resolved in SDS-PAGE and electrotransferred onto a nitrocellulose membrane as described previously [18].

For detection of the extracellular pool of fibronectin, the cells were seeded on 15 cm plates in a complete medium and cultured until 70% confluency was achieved. Next, the complete medium was replaced with the respective serum-free medium and the cells were cultured for an additional 24 h. The conditioned media were then collected, concentrated as described previously [35], and brought to the same protein concentration. Next, the resulting samples were resolved in SDS-PAGE and electrotransferred onto a nitrocellulose membrane as described previously [18].

Non-specific binding sites on the membranes were blocked with respective blocking reagents and the membranes were incubated with primary antibodies followed by respective secondary antibodies conjugated with HRP. Next, immunoblots were developed with enhanced chemiluminescence (ECL) using either X-ray films or the ChemiDoc MP System (Bio-Rad, Hercules, CA, USA). The antibodies and blocking/incubation solutions used in western blotting are listed in Supplementary Table S2. 

For densitometric analyses immunoblots were developed using an ECL substrate kit and subsequently scanned with the ChemiDoc MP System (Bio-Rad, Hercules, CA, USA). Densitometry was performed using ImageLab software (ver. 6.0, Bio-Rad, Hercules, CA, USA). At least three independent experiments were conducted for each quantified protein. The results were normalized to the total protein content (judged by densitometric analysis of Ponceau S stainings).

Lectin blotting was performed as described previously [36], except that biotinylated lectins (GSL II or VVL, either diluted 1:100) were detected using HRP-conjugated streptavidin (Vector Laboratories, Burlingame, CA, USA; diluted 1:50,000) followed by ECL detection using the ChemiDoc MP System (Bio-Rad, Hercules, CA, USA).

### 2.7. SYBR Green-Based Quantitative PCR

Total RNA was extracted from ~3 × 10^6^ cells using ExtractMe Total RNA Kit (Blirt, Gdańsk, Poland); cDNA was synthesized using LunaScript RT Supermix Kit (New England Biolabs, Ipswich, MA, USA). For this purpose, 0.75 μg of purified total RNA was mixed with 3 μL of LunaScript RT Supermix (5X) and supplemented with nuclease-free water up to a total volume of 15 μL. The obtained reaction mixture was incubated for 2 min at 25 °C (primer annealing), followed by 10 min at 55 °C (cDNA synthesis) and 1 min at 95 °C (heat inactivation).

To set up a qPCR experiment, 14 μL of freshly produced cDNA was mixed with 105 μL of the RT HS-PCR Mix SYBR A (A&A Biotechnology, Gdańsk, Poland) and 70 μL of MilliQ. Aliquots (18 μL) of the resulting master mix were pipetted into a 96-well PCR plate. Each qPCR assay (individual well on the plate) was completed with individual forward and reverse primers (1 μL of 10 μM solution each). The sequences of primers used in this study were sourced from the study of Jung et al. [37] and are listed in Supplementary Table S3.

The 96-well plate with complete 20 μL qPCR assays was sealed with foil, spun down in a centrifuge equipped with a 96-well plate swing-bucket rotor to remove any potential air bubbles, and subjected to a PCR using a LightCycler 96 instrument (Roche, Basel, Switzerland). The amplification was initiated by a 180 s denaturation at 95 °C and included 45 thermal cycling steps (denaturation at 95 °C for 15 s; primer annealing at 60 °C for 30 s and extension at 72 °C for 20 s).

The homogeneity of the PCR products was confirmed by the analysis of the thermal denaturation curves and by electrophoresis in 3% agarose gel. To control for a potential contamination of cDNA with genomic DNA, negative (no-RT) controls were included.

Relative expression folds were calculated using the ΔΔCq method following Taylor et al. [38]. A housekeeping gene, glyceraldehyde-3-phosphate dehydrogenase (GAPDH; NM_002046.7), served as an arbitrarily chosen reference. Each assay was performed in three biological replicates. The expression levels of the tested genes in the knockout cells were presented as relative fold changes with respect to the expression levels of the wild-type, which were set to unity. 

### 2.8. Spontaneous Migration Assay

Spontaneous migration assay was performed as described previously [39]. Briefly, 250 cells were seeded in a complete medium into each well of the dedicated 96-well plate (IncuCyte ImageLock, Sartorius, Goettingen, Germany), and the plates were incubated in an IncuCyte Live Cell Analysis Imaging System (Sartorius, Goettingen, Germany). Series of plate images were collected from 0 to 72 h every 2 h. Collected images were analyzed with a Manual Tracking plugin (ImageJ, F. Cordelieres, Institute Curie, Paris, France).

### 2.9. In Vitro Invasion Assay

The assay was performed as described previously [40]. Briefly, after 24 h of serum starvation, the cells were seeded in a serum-free medium onto transwell filters (Becton Dickinson, Franklin Lakes, NJ, USA) coated with Matrigel at a concentration of 1 mg/mL (Becton Dickinson, Franklin Lakes, NJ, USA); 20% fetal bovine serum (FBS) was used as a chemoattractant. After 24 h, the cells that traversed the Matrigel layer to the lower side of the insert membrane were fixed and stained with Hoechst 33342 (Thermo Fisher Scientific, Waltham, MA, USA), and nuclei were counted using a fluorescent microscope.

### 2.10. Image Processing

The confocal and light microscopy images were processed using ImageJ software (F. Cordelieres, Institute Curie, Paris, France).

### 2.11. Statistical Analysis

Statistical parameters including data plotted (mean ± SD) and *p*-values assignment; statistical tests used are detailed in figure legends. All statistical analyses were performed using GraphPad Prism 6 (GraphPad Software, Inc., San Diego, CA, USA).

## 3. Results

### 3.1. Wild-Type and SLC35A2-Deficient MDCK Cells Display Distinct Morphologies

**Figure 1:**
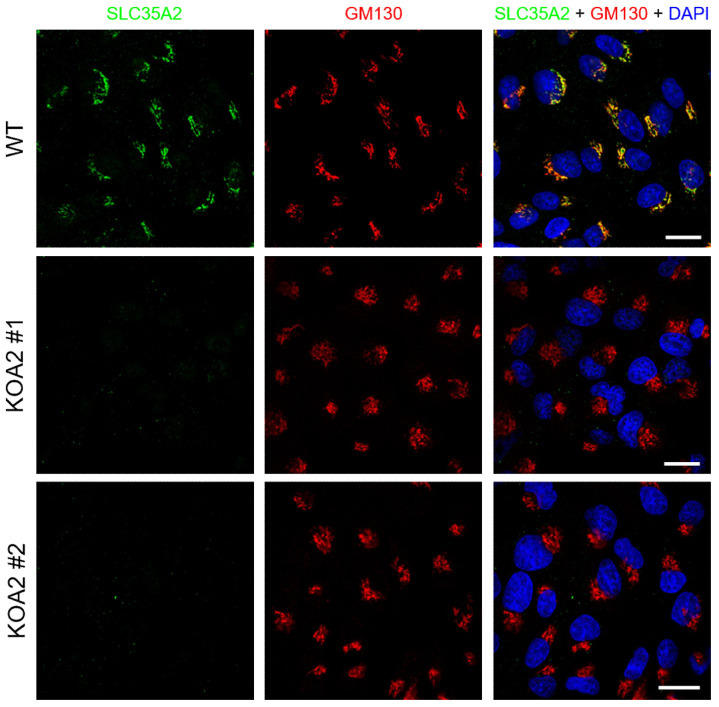
Confirmation of *SLC35A2* knockout in the selected MDCK clones by immunofluorescence staining. SLC35A2 is shown in green, GM130 is shown in red, and cell nuclei are shown in blue. Scale bar corresponds to 20 µm.

In the first stage of our study, several putative *SLC35A2* knockout clones were generated using the CRISPR/Cas9 strategy. We selected two clones for further analyses. To confirm disruption of the *SLC35A2* gene, we immunostained the wild-type and knockout cells with a rabbit antibody targeting the C-terminus of SLC35A2. To visualize the Golgi complex, the cells were counterstained with an antibody specific for the *cis* Golgi marker GM130. In the wild-type cells, we observed perinuclear signals that strongly colocalized with the Golgi marker, whereas no such signals could be detected in the knockout clones (Figure 1). Therefore, we assumed that the *SLC35A2* gene was successfully disrupted by the CRISPR-Cas9 approach. 

**Figure 2:**
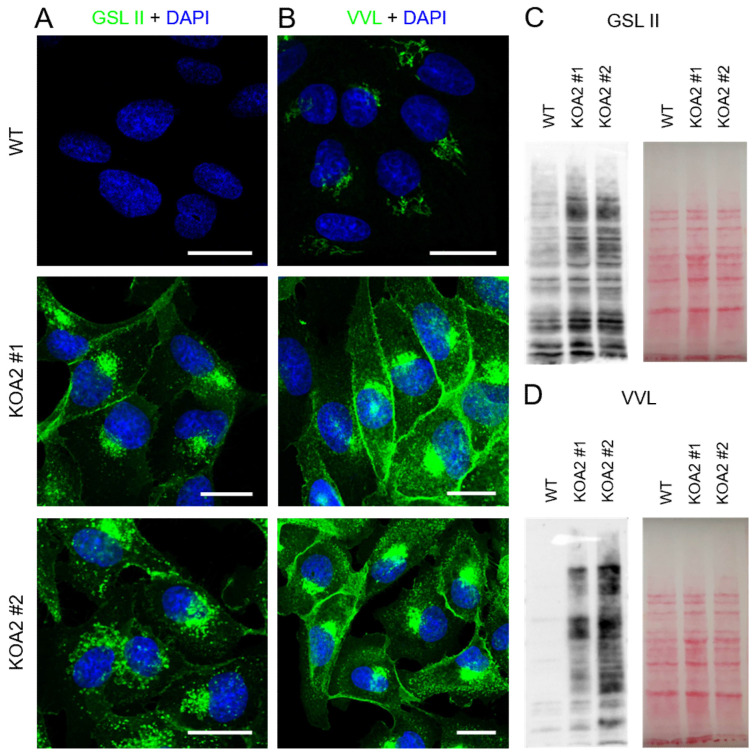
SLC35A2-deficient MDCK cells have impaired galactosylation. (**A**,**B**) Lectin stainings of cellular glycoconjugates using GSL II (**A**) and VVL (**B**). Lectin-stained glycoconjugates are shown in green and cell nuclei are shown in blue. Scale bar corresponds to 20 µm. (**C**,**D**) Lectin blotting analyses of cellular glycoproteins using GSL II (**C**) and VVL (**D**). Ponceau S stainings are presented alongside to demonstrate equal loading.

SLC35A2 depletion is expected to result in the synthesis of galactose-deficient glycoconjugates due to a shortage of UDP-galactose supply to the Golgi lumen. This leads to the exposure of the underlying sugars, i.e., *N*-acetylglucosamine in the case of N-glycans and *N*-acetylgalactosamine in the case of mucin-type O-glycans, which in turn enhances binding of certain lectins to the resulting oligosaccharides. We showed that glycoconjugates synthesized by the *SLC35A2* knockout cells displayed a greatly enhanced reactivity with *Griffonia simplicifolia* lectin II (GSL II) specific for *N*-acetylglucosamine (Figure 2A), as well as *Vicia villosa* lectin (VVL) specific for *N*-acetylgalactosamine (Figure 2B), which confirmed the loss of galactose from glycoconjugates. Similar results were obtained using lectin blotting (Figure 1C,D).

**Figure 3:**
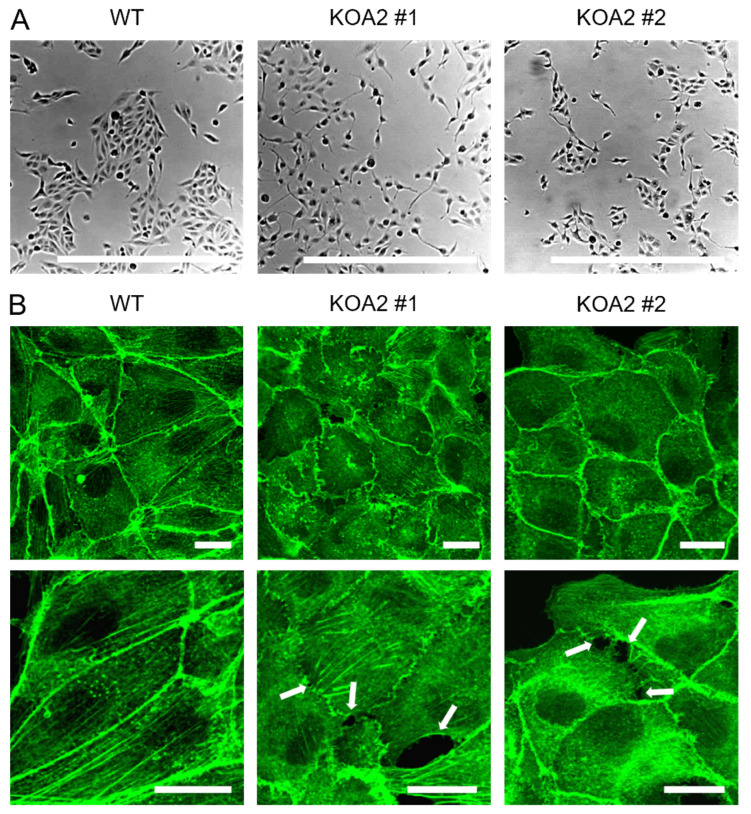
Distinct morphologies of the wild-type and SLC35A2-deficient MDCK cells. (**A**) General morphology of the wild-type cells and *SLC35A2* knockout clones showed by transmitted light micrographs. Scale bar corresponds to 500 µm. (**B**) The results of F-actin staining using fluorophore-conjugated phalloidin. The upper panel contains images obtained at a lower magnification, whereas the lower panel contains images obtained at a higher magnification. The sites of local disruption of cell–cell contacts in the knockout clones are indicated with arrows. Scale bar corresponds to 20 µm.

While working with the cells, we quickly realized that the *SLC35A2* knockout clones displayed an entirely different appearance than the wild-type cells. In sharp contrast to the cobblestone characteristics of the latter, the knockouts adopted an elongated, spindle-shaped morphology with impaired cell–cell adhesion (Figure 3A). To better visualize details of cellular morphology, we stained F-actin in the wild-type and knockout cells. The wild-type cells were of regular, polygon-like shapes with smooth edges, whereas the shapes of the *SLC35A2* knockout cells were irregular and their edges were jagged with discontinuities along the boundaries between adjacent cells (Figure 3B). This confirmed that cell–cell adhesion in the *SLC35A2* knockouts is compromised, although the overall F-actin organization was not perturbed. The remarkable changes in cellular morphology of the knockouts encouraged us to hypothesize that SLC35A2 deficiency promotes an EMT-like phenotype in MDCK cells. Therefore, we next undertook a series of experiments to verify whether the *SLC35A2* knockout clones indeed underwent EMT.

### 3.2. SLC35A2 Deficiency Downregulates E-Cadherin Expression 

**Figure 4:**
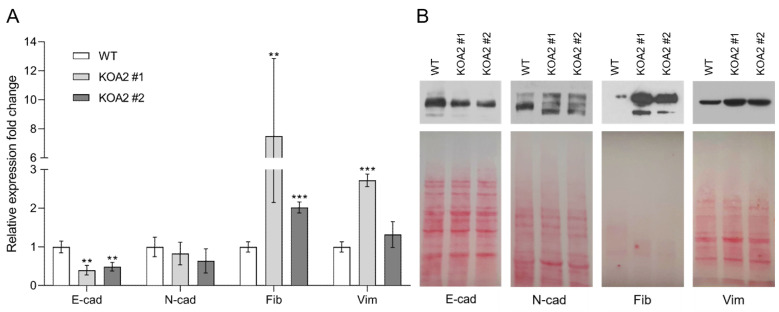
The effect of SLC35A2 deficiency on the transcript (**A**) and protein (**B**) levels of the selected EMT markers. (**A**) Quantitative analysis of the expression levels of the selected genes in the wild-type and SLC35A2-deficient MDCK cells. The heights of the columns represent relative fold changes of the expression in the knockouts with respect to the wild-type. Error bars represent ±1 SD. Asterisks indicate statistically important differences between wild-type cells and individual clones. The significance level was set at *p* ≤ 0.01 (**) and *p* ≤ 0.001 (***). Data were analyzed using unpaired *t*-test. (**B**) Western blotting analysis of intracellular (E-cadherin, N-cadherin, vimentin) and extracellular (fibronectin) protein levels. Ponceau S stainings are presented alongside to demonstrate equal loading. Representative data out of several independent experiments are shown. Abbreviations: E-cad, E-cadherin; N-cad, N-cadherin; Fib, fibronectin; Vim, vimentin.

The loss of E-cadherin is one of the key events accompanying EMT. Therefore, we compared the transcript and protein levels of this cell–cell adhesion molecule between the wild-type and SLC35A2-deficient cells. We found that the levels of E-cadherin transcript decreased more than two-fold in both knockout clones as compared with the wild-type cells (Figure 4A). On the protein level, both clones displayed a significant depletion of E-cadherin (Figure 4B). Typically, the loss of E-cadherin is accompanied by increased expression of N-cadherin (the so-called “cadherin switch”). However, in the case of SLC35A2-deficient cells, we did not observe any upregulation of N-cadherin expression (Figure 4A,B). We also noticed that N-cadherin produced by the knockouts migrated faster in SDS-PAGE than its wild-type counterpart, most likely due to defective glycosylation, as N-cadherin is a glycoprotein. Overall, we concluded that the loss of SLC35A2 activity triggers a significant downregulation of E-cadherin expression, both on the genetic and protein levels, but it does not upregulate N-cadherin expression.

### 3.3. SLC35A2 Deficiency Upregulates Fibronectin Expression 

The upregulation of fibronectin expression is another typical hallmark of an EMT. It is also a part of the fibrotic response, a phenomenon that involves an enhanced synthesis, secretion, and deposition of ECM proteins, which in turn adversely affects organ function.

We found that fibronectin expression was significantly increased in both knockout clones as compared to the wild-type cells, i.e., ~2- and ~8-fold for clones #2 and #1, respectively (Figure 4A). Even though one of the three biological repetitions that yielded ~16-fold increase and affected the mean and SD of clone #1 is disregarded, the mean expression level of fibronectin in clone #1 is still ~5-fold higher than in the wild-type.

We next checked whether the upregulation of fibronectin expression in the *SLC35A2* knockouts would translate into an increased secretion of this protein. We found that the conditioned serum-free media of the knockouts contained remarkably more fibronectin than the corresponding medium of the wild-type (Figure 4B). We therefore concluded that the SLC35A2-deficient MDCK cells express and secrete remarkably more fibronectin than the wild-type cells, which is indicative of fibrotic response.

### 3.4. SLC35A2 Deficiency Triggers Reorganization of the Vimentin Cytoskeleton

**Figure 5:**
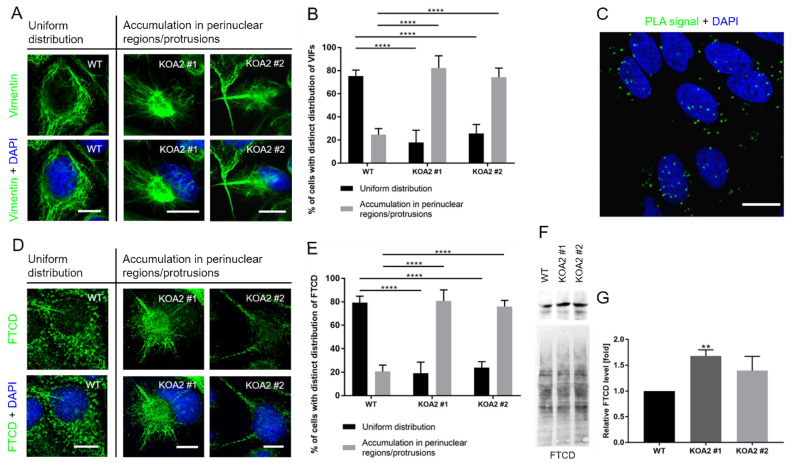
SLC35A2 deficiency affects organization of VIFs. (**A**,**D**) Representative images of distinct vimentin (**A**) and FTCD (**D**) staining patterns. VIFs (**A**) and FTCD (**D**) are shown in green and cell nuclei are shown in blue. Scale bar corresponds to 10 µm. The way of data presentation was inspired by [41]. (**B**,**E**) Quantitative analysis of the percentage of cells with distinct distribution of VIFs (**B**) and FTCD (**E**) in the wild-type and *SLC35A2* knockout cells. Data are presented as mean ± SD (6–9 images and 100 cells were analyzed for each cell line). Asterisks indicate statistically important differences between wild-type cells and individual clones. The significance level was set at *p* ≤ 0.0001 (****). Data were analyzed using ordinary one-way ANOVA with post hoc (Tukey’s multiple comparisons) test. (**C**) Results of in situ PLA analysis of proximity between FTCD and vimentin in the wild-type cells. PLA signals are shown in green and cell nuclei are shown in blue. Scale bar corresponds to 20 µm. (**F**) Western blotting analysis of the FTCD level. Ponceau S staining is presented alongside to demonstrate equal loading. Representative data out of several independent experiments are shown. (**G**) Densitometric analysis of the FTCD level (*n* = 3). Data are presented as mean ± SD. Asterisks indicate statistically important differences between wild-type cells and individual clones. The significance level was set at *p* ≤ 0.01 (**). Data were analyzed using ordinary one-way ANOVA with post hoc (Tukey’s multiple comparisons) test.

Vimentin, an intermediate filament protein, is another typical mesenchymal marker and its upregulation often accompanies EMT. Therefore, we examined vimentin level and its subcellular distribution in the wild-type and *SLC35A2* knockout MDCK cells. Our qPCR analysis demonstrated an above 2.5-fold upregulation of vimentin expression in clone #1, whereas clone #2 displayed only a slight and statistically insignificant increase in the vimentin transcript level (Figure 4A). Elevated vimentin expression was further confirmed by immunoblotting (Figure 4B). We also observed alterations in the distribution of vimentin intermediate filaments (VIFs). Namely, in the majority of wild-type cells, VIFs were evenly distributed in the cytoplasm, whereas in the majority of knockout cells they were retracted to perinuclear regions, where they often formed cage-like structures or accumulated in cell protrusions (Figure 5A,B and Figure S1). In conclusion, the *SLC35A2* knockouts displayed an increased level of vimentin and an altered subcellular distribution of VIFs.

Next, we examined the level and intracellular distribution of FTCD in the wild-type and *SLC35A2* knockout cells. This protein has drawn our attention due to its reported association with vimentin [42]. Specifically, FTCD has been shown to integrate with VIFs into chimeric fibers, triggering rearrangements of the vimentin cytoskeleton. We therefore investigated whether the alterations of the vimentin cytoskeleton observed in the SLC35A2-deficient cells would be accompanied by similar changes in the distribution of FTCD.

First, we have demonstrated that FTCD and vimentin are in a close proximity within the wild-type MDCK cells using an in situ PLA assay. As shown in Figure 5C, multiple PLA signals with a uniform distribution could be detected in the wild-type cells when an anti-FTCD antibody was combined with an anti-vimentin antibody.

Having found that FTCD is in a close proximity to vimentin in MDCK cells, we next immunostained FTCD in the wild-type and *SLC35A2* knockout cells. In the majority of wild-type cells, FTCD was uniformly distributed in the cytoplasm and showed a predominantly punctuated pattern with rarely occurring filaments (Figure 5D,E and Figure S2). However, in the majority of knockout cells, a significant subset of FTCD could be clearly assigned to filamentous structures that were present in the perinuclear regions or cell protrusions, i.e., in the areas where VIFs were shown to accumulate.

We also found that both knockout clones produced more FTCD than the wild-type, as shown by western blotting (Figure 5F), although in the case of clone #2, the increase in the FTCD level was not statistically significant (Figure 5G). We therefore concluded that FTCD may mediate the reorganization of VIFs triggered by SLC35A2 depletion. 

### 3.5. SLC35A2 Deficiency Triggers Golgi Compation

Many pathological conditions are associated with structural and functional changes in the Golgi structure. For example, Golgi fragmentation has been observed in numerous tumors (a so-called “onco-Golgi”) [43]. On the other hand, an EMT-driven pro-metastatic Golgi compaction with improved ribbon linking and cisternal stacking has also been reported [44].

**Figure 6:**
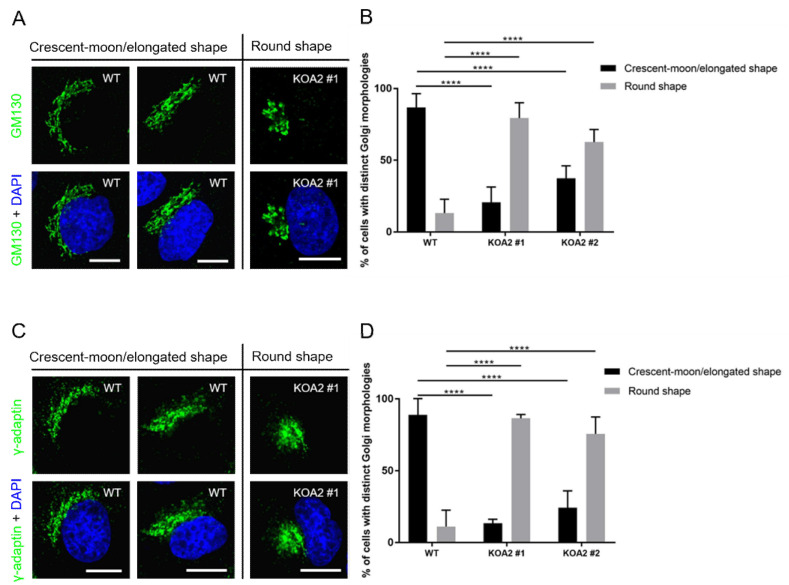
SLC35A2 deficiency triggers Golgi compaction. (**A**,**C**) Representative images of distinct Golgi morphologies. GM130 (**A**) and γ-adaptin (**C**) are shown in green and cell nuclei are shown in blue. Scale bar corresponds to 10 µm. The way of data presentation was inspired by [41]. (**B**,**D**) Quantitative analysis of the percentage of cells with distinct shapes of the *cis* Golgi compartment (**B**) and TGN (**D**) in the wild-type and *SLC35A2* knockout cells. Data are presented as mean ± SD (7–8 images and 104–117 cells were analyzed for each cell line). Asterisks indicate statistically important differences between wild-type cells and individual clones. The significance level was set at *p* ≤ 0.0001 (****). Data were analyzed using ordinary one-way ANOVA with post hoc (Tukey’s multiple comparisons) test.

Therefore, we have examined the Golgi morphology in the wild-type and *SLC35A2* knockout MDCK cells by immunostaining of GM130. We found that the Golgi apparatus of the majority of the wild-type cells displayed a crescent-moon/elongated shape, while in the majority of the knockout cells, it has become round (Figure 1, Figure 6A,B and Figure S3).

Next, we checked whether similar alterations could be observed for a different Golgi compartment, i.e., the *trans* Golgi network (TGN). For this purpose, we immunostained γ-adaptin, a component of the adaptor protein 1 (AP-1) complex which is present on the clathrin-coated membranes of the TGN [45]. We found that the alterations of the TGN morphology in the *SLC35A2* knockout cells mimic those observed in the case of the *cis* Golgi compartment (Figure 6C,D and Figure S4). We therefore concluded that the loss of SLC35A2 in MDCK cells triggers compaction of the Golgi complex, which has been previously linked with the EMT [44]. 

### 3.6. SLC35A2 Deficiency Enhances Cell Migration and Invasiveness

**Figure 7:**
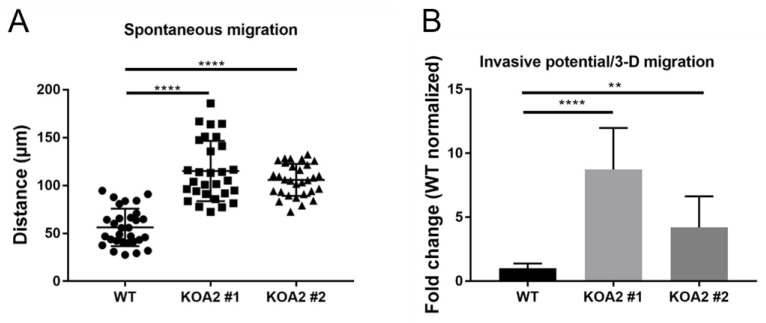
SLC35A2 deficiency enhances migratory and invasive properties of MDCK cells. (**A**) Results of the spontaneous migration assay. The cells were seeded into the wells of a 96-well ImageLock plate and monitored for 72 h with the IncuCyte system. Next, trajectories of single cells (*n* = 30) were plotted and distance traveled by the moving cells was assessed. (**B**) Results of the in vitro invasion assay (*n* = 10). Data are expressed as mean ± SD. Asterisks indicate statistically important differences between wild-type cells and individual clones. The significance level was set at *p* = 0.0095 (**) and *p* = 0.0001 (****). Data presented in both panels were analyzed using ordinary one-way ANOVA with post hoc (Dunnett’s multiple comparison) test.

Cells undergoing EMT tend to acquire enhanced migratory and invasive properties [30]. Therefore, we examined the wild-type and SLC35A2-deficient MDCK cells also in these terms. First, we performed a spontaneous migration assay. In this experiment, 2-D cell cultures were continuously monitored for 72 h, the trajectories of individual cells were recorded, and the distances they had travelled were calculated. We demonstrated that both knockout clones travelled significantly longer distances than the wild-type cells (Figure 7A and Supplementary Videos S1–S3). Thus, we concluded that SLC35A2 deficiency enhances the motility of MDCK cells.

Next, we performed an in vitro invasion assay, in which the ability of cells to migrate through both Matrigel (a substance that mimics the ECM) and the pores of the transwell filter towards a chemoattractant was investigated. In this type of assay, cells are allowed to migrate in 3-D, which reflects their ability to penetrate the surrounding environment, i.e., to invade. We found that the *SLC35A2* knockouts had significantly greater ability to migrate in 3-D compared with the wild-type (Figure 7B), although this effect was more pronounced in the case of clone #1 compared with clone #2. Therefore, we concluded that the *SLC35A2* knockout MDCK cells gain migratory and invasive properties, which is another indication of a shift towards a mesenchymal phenotype resulting from SLC35A2 depletion.

### 3.7. SLC35A2 Deficiency Upregulates Expression of Selected EMT-Activating Transcription Factors

EMT is orchestrated by a number of EMT-activating transcription factors (EMT-TFs) which mainly are members of Snail, Twist, and ZEB families [46]. Therefore, to gain an insight into the mechanism responsible for driving EMT in the SLC35A2-deficient MDCK cells, we examined protein levels of selected EMT-TFs. 

**Figure 8:**
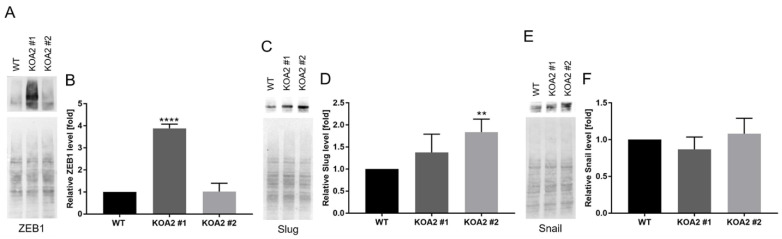
*SLC35A2* knockout upregulates expression of selected EMT-TFs in MDCK cells. (**A**,**C**,**E**) Western blotting analysis of the levels of ZEB1 (**A**), Slug (**C**), and Snail €. Ponceau S stainings are presented alongside to demonstrate equal loading. Representative data out of several independent experiments are shown. (**B**,**D**,**F**) Densitometric analysis of the levels of ZEB1 ((**B**), *n* = 3), Slug ((**D**), *n* = 4), and Snail ((**F**), *n* = 3). Data are presented as mean ± SD. Asterisks indicate statistically important differences between wild-type cells and individual clones. The significance level was set at *p* ≤ 0.01 (**) and *p* ≤ 0.0001 (****). Data were analyzed using ordinary one-way ANOVA with post hoc (Tukey’s multiple comparisons) test.

First, we investigated the level of ZEB1. We focused on this EMT-TF as its ectopic expression in MDCK cells was shown to cause Golgi compaction [44], which was evident in our knockouts (Figure 6). We found that the level of ZEB1 was ~4-fold elevated in clone #1 (Figure 8A,B). Surprisingly, however, we did not observe any increase in its level in the case of clone #2. 

Therefore, to unravel the molecular basis for EMT induction in the latter, we also checked the levels of Slug and Snail. As shown in Figure 8C,D, the level of Slug was nearly two-fold elevated in clone #2. In clone #1, its relative amount also tended to be increased, but this effect was not statistically significant. On the other hand, the level of Snail was not markedly changed in any of the clones (Figure 8E,F). We therefore concluded that in individual *SLC35A2* knockout clones EMT was driven by distinct TFs: in the case of clone #1, the EMT-TF that became upregulated was ZEB1, whereas in the case of clone #2, it was Slug.

## 4. Discussion

**Figure 9:**
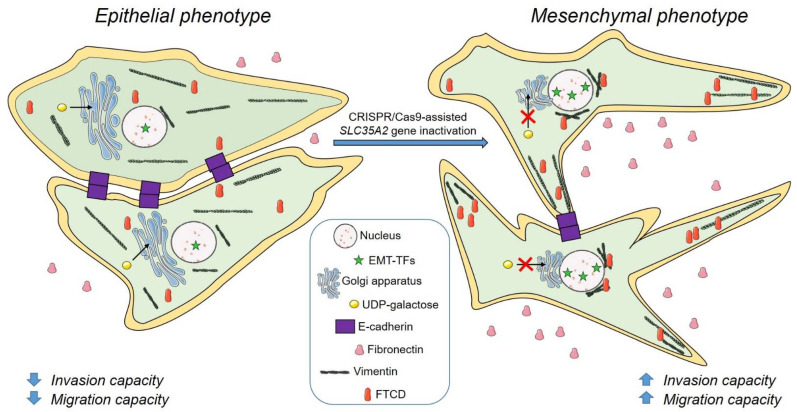
A summary of EMT-related changes in MDCK cells occurring upon the *SLC35A2* gene knockout. MDCK cells deficient in the SLC35A2 activity display increased levels of selected EMT-activating transcription factors (EMT-TFs), i.e., ZEB1/Slug, downregulation of E-cadherin expression, upregulation of fibronectin expression, as well as elevated level of its secretion, upregulation of vimentin expression, reorganization of VIFs, altered subcellular distribution of FTCD, Golgi compaction, and increased cell motility and invasiveness. The figure was assembled with the Servier Medical Art by Servier, licensed under a Creative Commons Attribution 3.0 Unported License.

In this work, we have demonstrated that knocking out the *SLC35A2* gene encoding the Golgi UDP-galactose transporter induces several mesenchymal features in the MDCK cell line without any additional stimulation towards an EMT. The EMT-related changes triggered by the SLC35A2 deficiency are depicted in Figure 9. To our best knowledge, this is the first report showing that knocking out a gene encoding a protein with a nucleotide sugar transporting activity promotes an EMT-like phenotype in non-malignant epithelial cells. 

Several functional links between other Golgi-resident, glycosylation-related proteins and EMT features have been established so far. T-synthase (C1GalT1) is the sole enzyme that attaches galactose to *N*-acetylgalactosamine during the biosynthesis of mucin-type O-glycans [47]. A knockout of the *C1GALT1* gene activated EMT process in human colorectal cancer cell line HCT116 [48]. TMEM165 is a putative ion transporter that regulates H^+^/Ca^2+^/Mn^2+^ homeostasis and pH in the Golgi apparatus [49]. The loss of TMEM165 activity triggers alterations in terminal glycosylation and causes a subtype of a CDG [50]. Knocking out the *TMEM165* gene in human invasive breast cancer cell line MDAMB231 attenuated malignant properties of the cells, whereas its overexpression triggered a more mesenchymal phenotype [51]. SLC35A3 is a multitransmembrane Golgi-resident protein with a putative UDP-*N*-acetylglucosamine transporting activity [52]. It shares a significant homology with SLC35A2, and both proteins were shown to interact [12,13]. In a recent study, a knockout of the *SLC35A3* gene in human cervical cancer cell line HeLa enhanced cell spreading while suppressing cell migration and proliferation [53]. However, it should be emphasized that all these studies were performed using cancer cells, while no similar reports are available for non-malignant cell lines.

Mechanistically, the *SLC35A2* knockout triggered upregulation of expression of selected EMT-TFs in MDCK cells. Interestingly, distinct EMT-TFs were elevated in different clones (ZEB1 in clone #1 and Slug in clone #2). This suggests that there is more than one mechanism through which EMT can be induced by SLC35A2 depletion. Although ectopic expression of ZEB1 was shown to cause Golgi compaction [44], other EMT-TFs were not investigated in these terms. Our results demonstrate that Slug may also be implicated in the EMT-associated Golgi compaction. The EMT-like phenotype was somewhat milder in clone #2 comparing with clone #1, which was reflected by the lack of statistically significant increases in the levels of vimentin transcript and FTCD protein, less prominent Golgi compaction, and lower invasive potential. We hypothesize that the differences between these two clones result from distinct identities of the EMT-TFs that become upregulated and the extent to which their levels are elevated. Importantly, it has been shown that the genetic programs induced by distinct EMT-TFs in MDCK cells are not entirely the same [54].

An obvious question is how the SLC35A2 deficiency promotes mesenchymal traits in MDCK cells, i.e., what is the molecular background behind the upregulated expression of ZEB1/Slug in the knockout clones. What is the mechanistic link between the loss of a Golgi-resident NST and increased levels of EMT-TFs, i.e., nuclear proteins? Undoubtedly, further studies are necessary to address this puzzling question. It appears reasonable to assume that there are some secondary, as yet unidentified effects of the loss of SLC35A2 activity that affect the levels of ZEB1/Slug. An expected and well-documented outcome of the *SLC35A2* knockout is the impaired galactosylation of macromolecules. However, providing an explanation as to how the defective glycosylation might influence the levels of EMT-TFs may not be a trivial task. Nevertheless, based on our results, an interesting observation can be made that the Golgi-associated events can be both a trigger (depletion of SLC35A2 from the Golgi membranes) for and a consequence (Golgi compaction) of EMT. Thus, by inducing EMT, which in turn causes Golgi compaction, the SLC35A2 deficiency, in a way, comes full circle.

EMT has been shown to trigger some specific changes in glycosylation [33,55,56]. Therefore, it is possible that the initial glycophenotype of the *SLC35A2* MDCK knockouts is further potentiated or additionally modified by EMT. In other words, it is highly likely that the final glycophenotype of the SLC35A2-deficient MDCK cells is a synergistic effect of both defective glycosylation and EMT, which should be kept in mind when exploring and defining glycosylation patterns of *SLC35A2* knockouts in general.

The fact that non-malignant epithelial cells acquire mesenchymal features upon the *SLC35A2* knockout poses the possibility of pathological EMT occurring in the tissues and organs of SLC35A2-CDG patients. To date, very few studies addressed any putative links between the EMT and the pathophysiology and clinical manifestation of CDGs. Lecca et al. [57] have shown that the fibroblasts derived from patients with type I CDGs develop fibrotic response which is a part of the EMT-associated phenotype. However, it should be underlined that type I CDGs are associated with more severe defects in glycosylation (i.e., decreased N-glycosylation sites occupancy), while in the case of SLC35A2-CDG, which is a type II CDG, only terminal glycosylation of macromolecules is impaired which may be expected to be less detrimental to the cell function. We show that fibrotic response, here understood as an enhanced expression and secretion of fibronectin, is also produced by epithelial cells as a putative consequence of defective galactosylation caused by SLC35A2 depletion. The awareness of the possibility that EMT and/or fibrotic response occur in SLC35A2-CDG patients may be important for our understanding of the molecular basis of some CDG-associated pathologies such as organ fibrosis. It must be emphasized that when it comes to CDG patients, skin fibroblasts and leukocytes are the most often studied types of cells. Therefore, the hypothetical effect of pathological EMT could have been easily overlooked in the affected individuals.

To summarize, here we demonstrate that SLC35A2 depletion promotes an EMT-like phenotype in non-malignant MDCK cell line. We hypothesize that this non-physiological EMT may contribute to the pathogenesis of SLC35A2-CDG. Our findings show that galactosylation of macromolecules may be important for the maintenance of the epithelial state of the cells and point to a novel role for SLC35A2 as a gatekeeper of the epithelial phenotype.

## Data Availability

Not applicable.

## Supplementary Materials

The following supporting information can be downloaded at: https://www.mdpi.com/article/10.3390/cells11152273/s1, Table S1: List of reagents used in immunofluorescence and fluorescence stainings; Table S2: List of antibodies used in western blotting experiments; Table S3: List of primer sequences used in qPCR experiments. Figure S1: Images of the wild-type and *SLC35A2* knockout MDCK cells stained with anti-vimentin antibody; Figure S2: Images of the wild-type and *SLC35A2* knockout MDCK cells stained with anti-FTCD antibody; Figure S3: Images of the wild-type and *SLC35A2* knockout MDCK cells stained with anti-GM130 antibody; Figure S4: Images of the wild-type and *SLC35A2* knockout MDCK cells stained with anti-γ-adaptin antibody; Video S1: Continuous movement of the wild-type MDCK cells during the spontaneous migration assay; Video S2: Continuous movement of the *SLC35A2* knockout MDCK cells (clone #1) during the spontaneous migration assay; Video S3: Continuous movement of the *SLC35A2* knockout MDCK cells (clone #2) during the spontaneous migration assay.

## Abbreviations

ANOVA, analysis of variance; AP-1, adaptor protein 1; ATP, adenosine triphosphate; BSA, bovine serum albumin; CDG, congenital disorder of glycosylation; cDNA, complementary DNA; CRISPR, clustered regularly interspaced short palindromic repeats; crRNA, CRISPR RNA; DAPI, 4′,6-diamidino-2-phenylindole; DNA, deoxyribonucleic acid; ECL, enhanced chemiluminescence; ECM, extracellular matrix; EMT, epithelial-to-mesenchymal transition; EMT-TFs, EMT-activating transcription factors; ER, endoplasmic reticulum; FBS, fetal bovine serum; FTCD, formiminotransferase cyclodeaminase; GAG, glycosaminoglycan; GAPDH, glyceraldehyde-3-phosphate dehydrogenase; GDP, guanosine diphosphate; GSL II, *Griffonia simplicifolia* lectin II; GTP, guanosine triphosphate; HLH, helix-loop-helix; HRP, horseradish peroxidase; MDCK, Madin-Darby canine kidney; MEM, minimal essential medium; NST, nucleotide sugar transporter; PAGE, polyacrylamide gel electrophoresis; PBS, phosphate buffered saline; PHA-L, phytohemagglutinin-L; PLA, proximity ligation assay; PCR, polymerase chain reaction; RNA, ribonucleic acid; RT, reverse transcription/room temperature; SDS, sodium dodecyl sulfate; SLC35A2, solute carrier family 35 member A2; SLC35A3, solute carrier family 35 member A3; SLC35A5, solute carrier family 35 member A5; SLC35C1, solute carrier family 35 member C1; TFs, transcription factors; TGF-β, transforming growth factor beta; TGN, *trans* Golgi network; TMEM165, transmembrane protein 165; tracrRNA, trans-activating crRNA; UDP, uridine diphosphate; UGT, UDP-galactose transporter; VIF, vimentin intermediate filament; VVL, *Vicia villosa* lectin.
