# Supplementary material for: SLC35A2 Deficiency Promotes an Epithelial-to-Mesenchymal Transition-like Phenotype in Madin–Darby Canine Kidney Cells
Source: Cells. 2022 Jul 23;11(15):2273. doi: 10.3390/cells11152273 (PMC9331475; doi:10.3390/cells11152273)
Supplement: Supplementary file 1 [file cells-11-02273-s001.zip › cells-1727161-supplementary tabs and figs.pdf]

cells MDPI

Supplementary Materials

SLC35A2 Deficiency Promotes an Epithelial-to-Mesenchymal Transition-Like Phenotype in Madin-Darby Canine Kidney Cells

Magdalena Kot [1,†], Ewa Mazurkiewicz [1,†], Maciej Wiktor [2], Wojciech Wiertelak [2], Antonina Joanna Mazur [1], Andrei Rahalevich [2], Mariusz Olczak [2] and Dorota Maszczak-Seneczko [2,*]

[1] Department of Cell Pathology, Faculty of Biotechnology, University of Wroclaw, 14A F. Joliot-Curie St., 50-383 Wroclaw, Poland; magdalena.kot@uwr.edu.pl (M.K.); ewa.mazurkiewicz@uwr.edu.pl (E.M.); antonina.mazur@uwr.edu.pl (A.J.M.)
[2] Department of Biochemistry, Faculty of Biotechnology, University of Wroclaw, 14A F. Joliot-Curie St., 50-383 Wroclaw, Poland; maciej.wiktor@uwr.du.pl (M.W.); wojciech.wiertelak@uwr.edu.pl (W.W.); andrrag2000@gmail.com (A.R.); mariusz.olczak@uwr.edu.pl (M.O.)
* Correspondence: dorota.maszczak-seneczko@uwr.edu.pl
† These authors contributed equally to this work.

Table S1. List of reagents used in immunofluorescence and fluorescence stainings.

Reagent	Vendor	Dilution
Rabbit anti-SLC35A2 (HPA036087)	Sigma-Aldrich	1:100
Mouse anti-FTCD (C-1)	Santa Cruz Biotechnology	1:25
Mouse anti-vimentin (V9)	Sigma-Aldrich	1:500
Mouse anti-GM130 (35/GM130)	BD Biosciences	1:100
Mouse anti-γ-adaptin (100/3)	Sigma-Aldrich	1:500
Goat anti-mouse IgG Alexa Fluor Plus 555	Thermo Fisher Scientific	1:200-1:1000
Goat anti-rabbit IgG Alexa Fluor Plus 488	Thermo Fisher Scientific	1:1000
Phalloidin conjugated with Alexa Fluor 568	Thermo Fisher Scientific	1:100

Table S2. List of antibodies used in western blotting experiments. [1-2]Solutions used for blocking and incubation with both primary and secondary antibodies. 15% BSA in TBS with 0.2% Tween 20; 25% non-fat dried milk in PBS with 0.2% Tween 20.

Antibody	Vendor	Dilution
Rabbit anti-E-cadherin (24E10)[1]	Cell Signaling Technology	1:1000
Rabbit anti-N-cadherin (D4R1H)[1]	Cell Signaling Technology	1:500
Mouse anti-vimentin (V9)[2]	Sigma-Aldrich	1:10000
Mouse anti-FTCD (C-1)[1]	Santa Cruz Biotechnology	1:500
Mouse anti-fibronectin (1G10F9)[1]	Proteintech	1:1000
Rabbit anti-ZEB1[1]	Proteintech	1:1000
Rabbit anti-Slug (C19G7)[1]	Cell Signaling Technology	1:500
Rabbit anti-Snail (C15D3)[1]	Cell Signaling Technology	1:1000
Goat anti-mouse IgG-HRP[1/2]	Proteintech	1:5000-1:10000
Goat anti-rabbit IgG-HRP[1]	Sigma-Aldrich	1:5000-1:10000

Table S3. List of primer sequences used in qPCR experiments.

Protein name	Forward primer sequence	Reverse primer sequence
E-cadherin	5′-AAAACCCACAGCCTCATGTC-3′	5′-CACCTGGTCCTTGTTCTGGT-3′
N-cadherin	5′-CCCAAGACAAGCGACTAAGC-3′	5′-TGACAGCTGACCTGAGATGG-3′
Fibronectin	5′-GGTTTCCCATTATGCCATTG-3′	5′-TTCCAAGACATGTGCAGCTC-3′
Vimentin	5′-CCGACAGGATGTTGACAATG-3′	5′-TCAGAGAGGTCGGCAAACTT-3′
GAPDH	5′-AACATCATCCCTGCTTCCAC-3′	5′-GACCACCTGGTCCTCAGTGT-3′

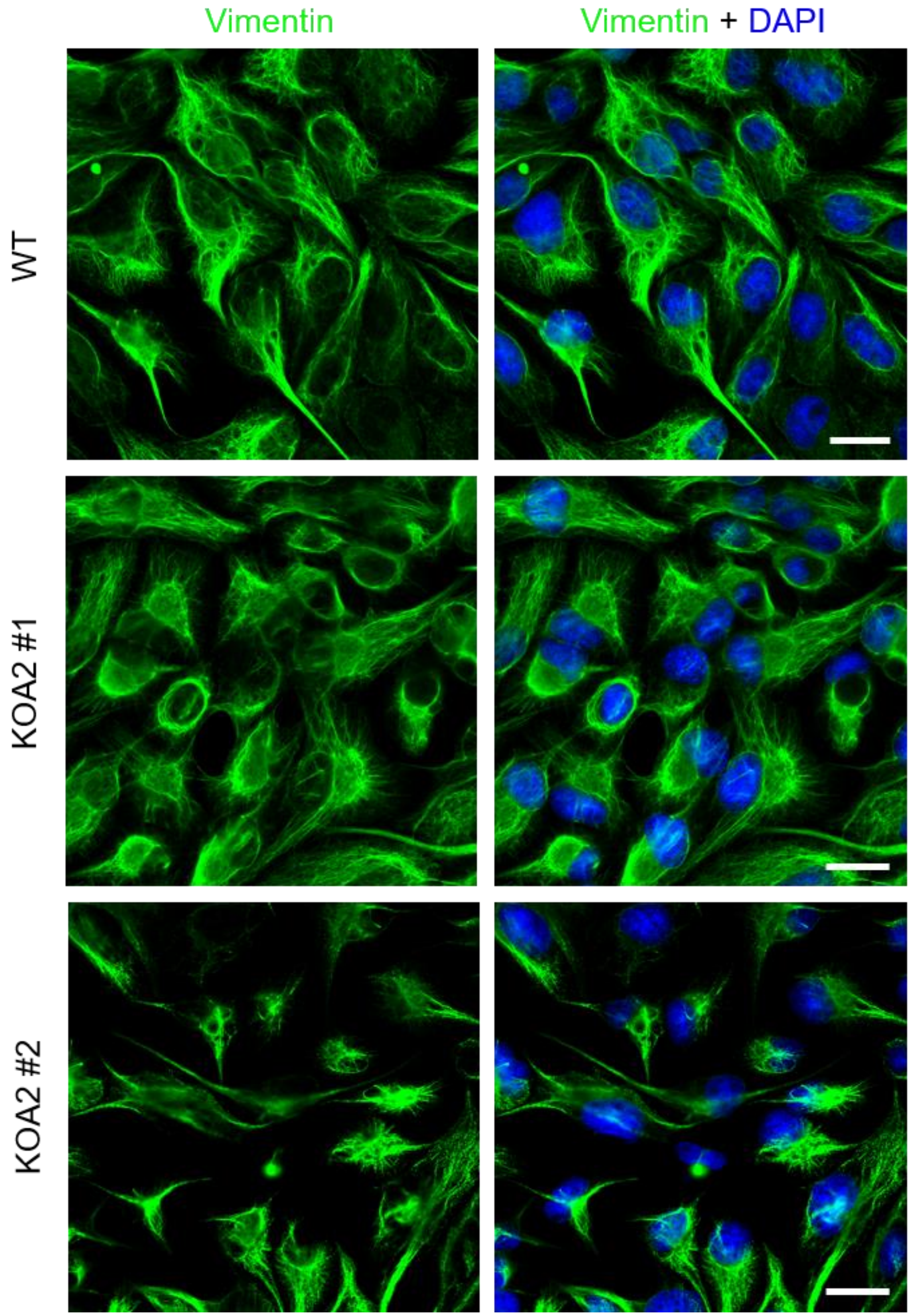

Figure S1. Images of the wild-type and *SLC35A2* knockout MDCK cells stained with anti-vimentin antibody. Vimentin is shown in green and cell nuclei are shown in blue. Scale bar corresponds to 20 µm.

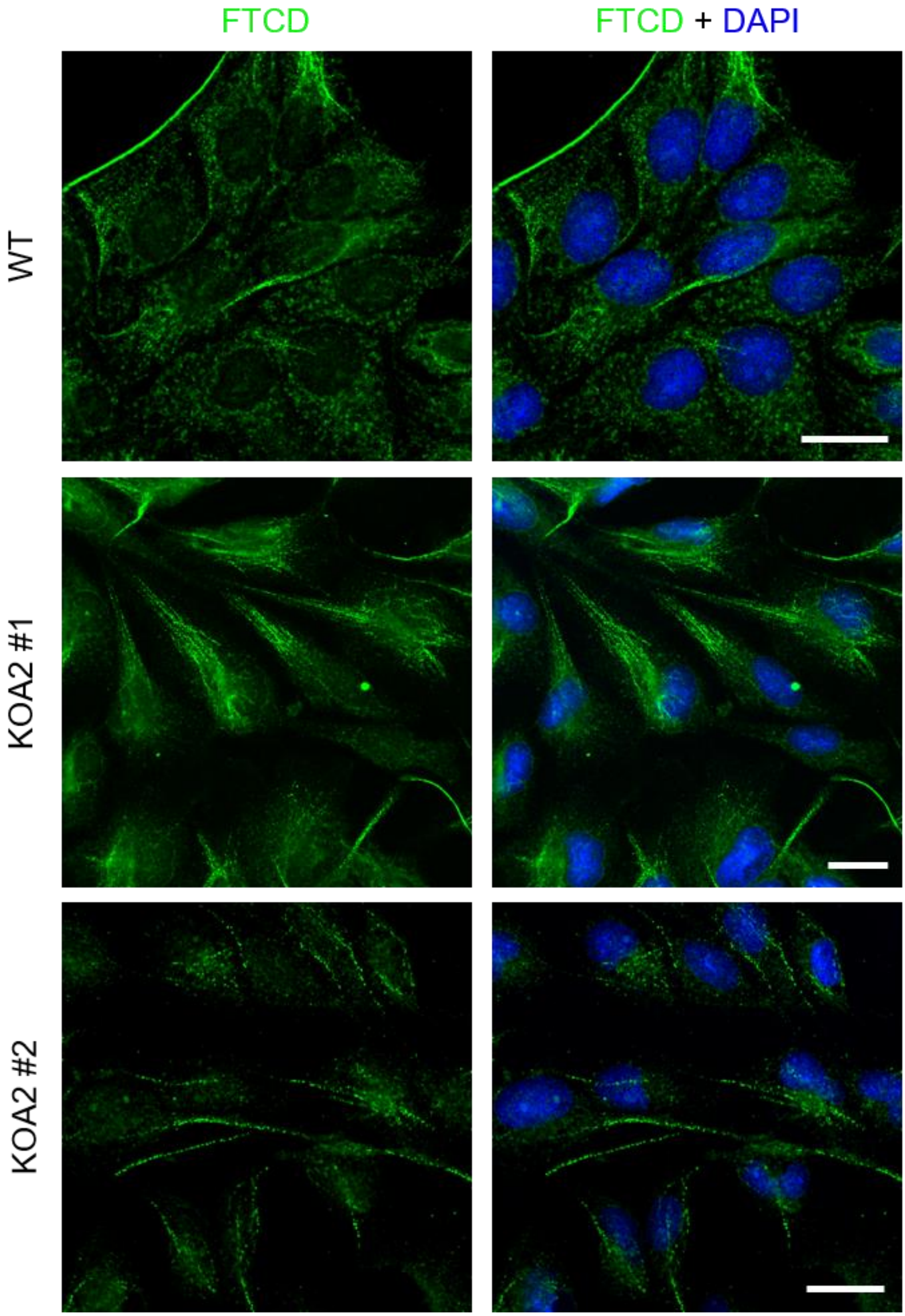

Figure S2. Images of the wild-type and *SLC35A2* knockout MDCK cells stained with anti-FTCD antibody. FTCD is shown in green and cell nuclei are shown in blue. Scale bar corresponds to 20 µm.

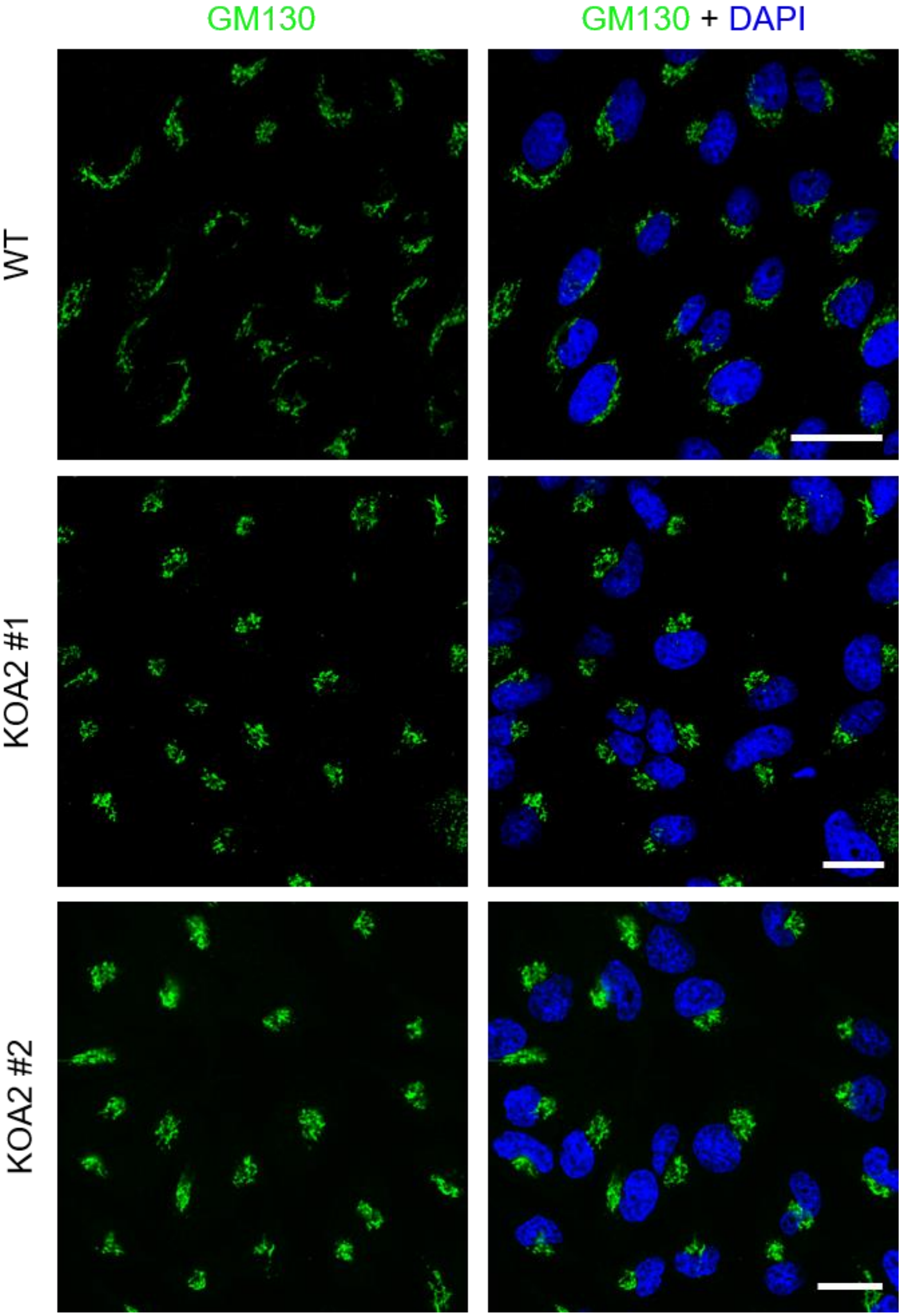

Figure S3. Images of the wild-type and *SLC35A2* knockout MDCK cells stained with anti-GM130 antibody. GM130 is shown in green and cell nuclei are shown in blue. Scale bar corresponds to 20 µm.

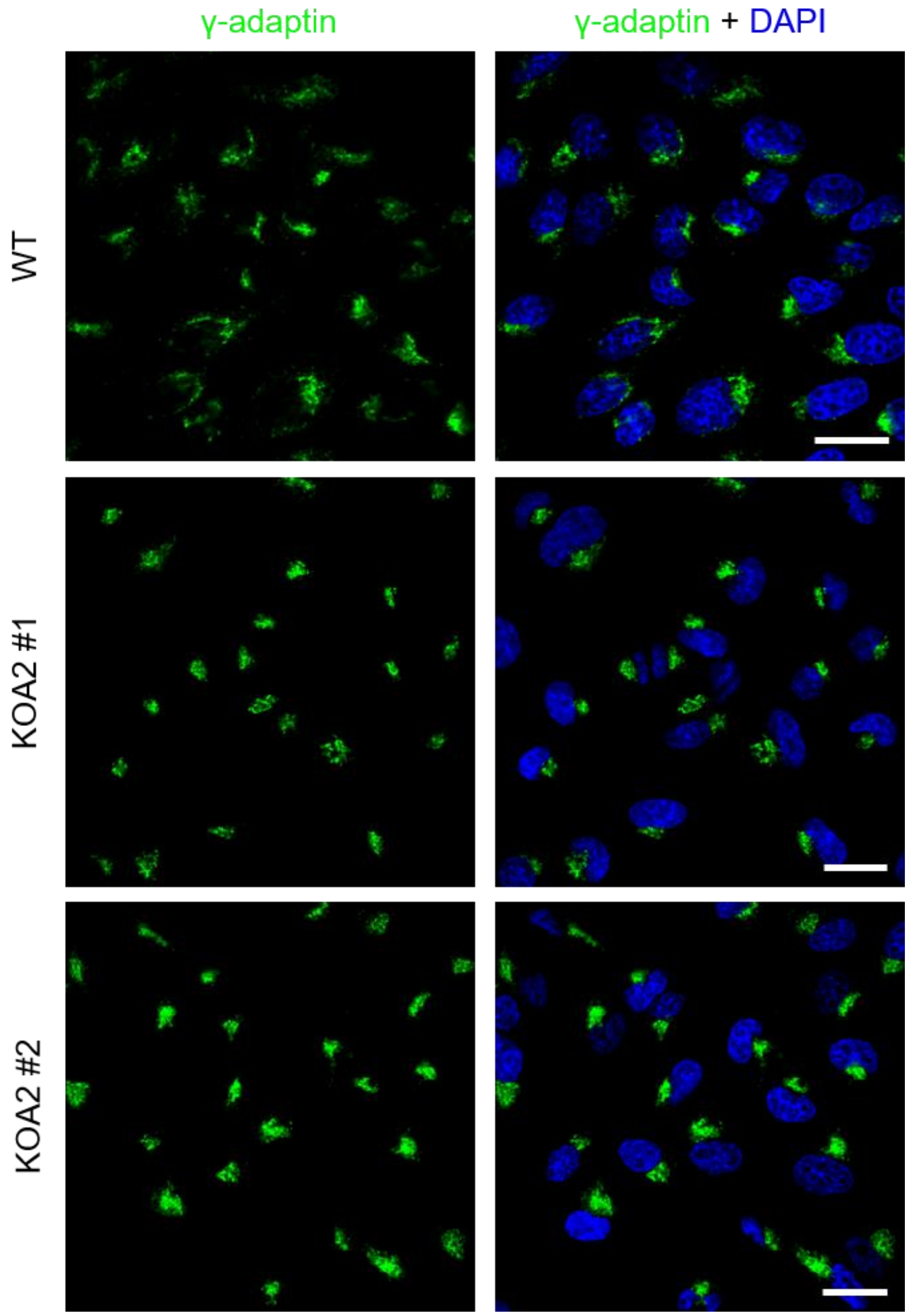

Figure S4. Images of the wild-type and *SLC35A2* knockout MDCK cells stained with anti-γ-adaptin antibody. γ-adaptin is shown in green and cell nuclei are shown in blue. Scale bar corresponds to 20 μm.
